# Attentional Window Set by Expected Relevance of Environmental Signals

**DOI:** 10.1371/journal.pone.0021262

**Published:** 2011-06-16

**Authors:** Marije van Beilen, Remco Renken, Erik S. Groenewold, Frans W. Cornelissen

**Affiliations:** 1 Laboratory for Experimental Ophthalmology & BCN NeuroImaging Center, School of Behavioural and Cognitive Neurosciences, University Medical Center Groningen, Groningen, The Netherlands; 2 Department of Neurology, University Medical Center Groningen, University of Groningen, Groningen, The Netherlands; Alexander Flemming Biomedical Sciences Research Center, Greece

## Abstract

The existence of an attentional window—a limited region in visual space at which attention is directed—has been invoked to explain why sudden visual onsets may or may not capture overt or covert attention. Here, we test the hypothesis that observers voluntarily control the size of this attentional window to regulate whether or not environmental signals can capture attention. We have used a novel approach to test this: participants eye-movements were tracked while they performed a search task that required dynamic gaze-shifts. During the search task, abrupt onsets were presented that cued the target positions at different levels of congruency. The participant knew these levels. We determined oculomotor capture efficiency for onsets that appeared at different viewing eccentricities. From these, we could derive the participant's attentional window size as a function of onset congruency. We find that the window was small during the presentation of low-congruency onsets, but increased monotonically in size with an increase in the expected congruency of the onsets. This indicates that the attentional window is under voluntary control and is set according to the expected relevance of environmental signals for the observer's momentary behavioral goals. Moreover, our approach provides a new and exciting method to directly measure the size of the attentional window.

## Introduction

In everyday life, our visual system is bombarded with signals that continuously attempt to draw our attention. For example, when on the road, we may need to look for street signs that indicate a change in the traffic situation amidst billboards that try to lure us to come over for a quick “bite”. While visually searching and exploring the world, observers continuously move their eyes, to direct the high-resolution section of their retinae to items of interest. During a fixation –the brief period during which the eyes do not move-a portion of the visual field can be scrutinized. Previous results [1] suggest that, once the fixated region or object has been scrutinized, information from lower resolution peripheral vision is used to plan a saccadic eye-movement to the next region or object (although these processes are presumably not independent [2] and the temporal relationship between object recognition and eye-movement may be less specific (e.g.[3])).

In addition to attending to the information that is relevant for the task at hand, it may be necessary to also consider other environmental signals that may provide unexpected but important information (onsets), for example those that indicate a dangerous situation. Also, the relevance of such unexpected visual information may vary, so we need to exert some kind of top-down control (e.g. inhibition of distraction) over the extent of attentional capture by unexpected visual onsets. These environmental distracters influence following eye-movements in their turn (bottom-up influence) [4].

### Attentional window

Previous research suggests that one of the mechanisms by which we dynamically control what information is allowed to capture attention, guide our gaze, and potentially drive our behaviour is an attentional window. The attentional window is a theoretical construct describing the limited region of visual space that attention can be paid to. The size of this region is derived by examining whether or not an event presented in visual space can attract or, in other words, capture, attention or gaze. In the latter case, the presence of a capture is determined by verifying whether an event evokes an eye-movement. This is also the approach we take in the present study.

The use of gaze recordings is justified by the large body of evidence that indicates that eye-movements are preceded by shifts of attention [5]. Thus, eye-movements executed during search tasks provide a pointer to the attentional processes underlying performance. A clear advantage of using eye-movement recordings is that these allow a much more refined spatial and temporal analysis of performance than does response time, a more global measure that is influenced by a variety of search and decision processes [6].

Theeuwes [7] introduced the attentional window concept and claimed that “top-down control over visual selection can be accomplished by endogenously varying the spatial attentional window”. Theeuwes used the existence of an attentional window to explain why salient color singletons fail to capture attention in some situations [8] while they do capture attention in others [9]. Belopolsky et al. [10] manipulated the size of the attentional window by requiring observers to detect either a global or a local shape prior to performing a search task. Participants' attention was captured more often when their attentional window was induced to be wide (global shape detection) than when it was induced to be narrow (local shape detection). Belopolsky et al. concluded that the size of the attentional window is an important factor in determining whether an irrelevant colour singleton will capture attention. This view integrated the earlier vision of bottom-up capture [7], [9], [11] and top-down regulation of unexpected singleton capture in search paradigms.

In a study by Yantis and Jonides [12], participants searched for a target while onsets (abruptly appearing stimuli) were presented in the periphery of their visual field. Prior to the start of a trial, the probable target location was indicated by a symbolic cue. When cues were completely valid (i.e. matching the actual location of the subsequent target), reaction times to target stimuli were unaffected by onsets while at lower cue congruency reaction times did increase. Yantis and Jonides explained their results in terms of a (voluntary) allocation of spatial attentional based on the cue pointing at the actual (congruent) or opposite (incongruent) side of consecutive onset location. A congruent cue made participants selectively focus attention at the cued location, inhibiting visual capture by an onset, whereas at lower cue congruency, spatial attention would be more diffuse, so that onset capture was not inhibited. This implies that visual capture of onsets is not a strongly automated process and could be under voluntary control.

### Serial search versus parallel search

The attentional window size may be dependent on the type of search task: i.e. an inefficient, serial search or a more efficient, parallel search (e.g. [13]). Serial search requires inspection of every possible target candidate one at a time, whereas during parallel search, all candidates can be evaluated in a single glimpse. Parallel search thus implies a wide spatial attentional window, whereas serial search implies a narrow attentional window [14], [15]. Automatic bottom-up processes are usually equated with parallel search and top-down processes with serial search [16] [15] [14].

### Top-down control?

Several studies suggest that voluntary control over attentional window size could be used to modulate attentional capture. If so, this could be a mechanism by which observers can regulate whether or not environmental signals will capture attention. A voluntary regulation of attentional window size would imply top-down guided processing of stimuli as opposed to a bottom-up adjustment to visual cues. Even though the idea of automatic bottom-up processing of capture events [7], [9], [11] has dominated the visual search literature, yet evidence for voluntary processes within visual search is now emerging from different perspectives and indicates that capture by unexpected environmental events can indeed be modulated in a top-down manner. For example, Braun and Sagi [17] reported that feature singletons in peripheral vision captured attention whilst participants performed a serial form-recognition search task in the centre of the screen. Even though the task implied a narrow attentional focus, peripheral capture does suggest a broad window. We believe that this implies that attentional window size can be manipulated voluntarily, to adjust to the momentary task set. Furthermore, Müller et al. [18] propose a top-down, voluntary weighting of stimulus salience during visual search tasks with onsets of different dimensions (e.g. colour, orientation). In addition to the studies that suggest a top-down regulation of visual capture by onsets, Belopolsky et al. [10] suggest that even though an attentional window may affect which onsets result in capture, capture within the attentional window would still be a bottom-up process.

### Current study

Here, we test the hypothesis that observers exert voluntary control over attentional capture by setting the size of the attentional window. Moreover, we expect them to do so depending on task-characteristics (as set by environmental factors and internal goals). In other words, we test the hypothesis that attentional window size (as derived from the likelihood of capture) is modulated by the expected relevance of environmental signals for the ongoing task.

Hence, we predict that the attentional window size will increase with onset congruency, even in an inefficient serial search task that is generally assumed to impose a narrow attentional focus. We tested this hypothesis by having participants' perform a search task, in which they could freely move their eyes over the screen (i.e. overt orienting). During the task, we presented abrupt onsets that indicated the position of the target with varying degrees of congruency. Cue congruency was reported to the participants prior to each block of trials. We used overt oculomotor capture (i.e. capture of gaze) as our primary measure of attentional capture. The eccentricities of the captured onsets were used to derive the attentional window size. The search task was designed such that we: (a) enabled participants to exhibit gaze behaviour, (b) could study attentional capture at eccentricities up to 20 degrees, (c) were able to study attentional capture during the search process itself, so as to minimize possible contamination by start up processes. Finally, by keeping stimulus and onset characteristics identical in all experimental conditions, we made sure that changes in attentional window size cannot be attributed to changes in onset saliency [19]. The novel contribution from the current study is (1) the direct measurement of the size of the attentional window and (2) the relationship between the size of the attentional window and participant's expectations about the relevancy of onsets.

## Methods

This study was approved by the ethical committee of the University Medical Centre Groningen, all participants gave written informed consent and were treated according to the declaration of Helsinki.

### Participants

Fourteen university students participated in the study. Age ranged from 20 to 25 years (mean age 22). Participants had normal or corrected-to-normal vision.

### Apparatus

Participants were seated in a darkened room at 85 cm from a CRT computer monitor (LaCie). The horizontal size of the monitor screen was 27 degrees of visual angle, and its vertical size was 20 degrees of visual angle. A chinrest stabilized the participant's head. A remote eyetracker (Eyelink 1000, SR Research, Mississauga, Ontario, Canada) was located below the screen to track eye position during the experiment at a sample rate of 1000 Hz. The experiment was written in MATLAB (Mathworks, Natick, MA, USA), using the Psychophysics and Eyelink Toolbox extensions [9], [10], [11] . The experimental room was dark except for the illumination provided by the monitor.

### Procedure

#### Experimental design

**Figure 1:**
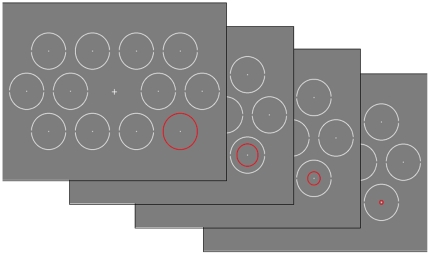
Participants searched for the single circle with one opening amidst 11 distracters with two openings. In this figure, the target is located at the top row, at the second position from the left. At a random moment during the trial, a red shrinking circle appeared abruptly on one of the circle's positions and disappeared after 100 ms.

During a trial, participants searched for a single circle with one opening (Target), amidst 11 circles with two openings (distracters) (see Figure 1). Circles were placed on the screen in a pattern such that the distances (centre to centre) between neighbouring circles was 5.8 deg. Consequently, the horizontal distance is 5.8 deg. Because neighbouring circles on different rows are vertically not aligned (see figure 1), the vertical distance between rows was 5.0 deg. The radius of the circles was 1.4 deg; the opening of the circles was 6 degrees of arc. Luminances of the (white) circles, (grey) background, and (red) abrupt onset were 111, 29 and 27 cd/m^2^, respectively.

Participants were required to report the target's open side (left or right) by pressing as fast as possible either the left or right arrow on the keyboard. During the trial, an abrupt onset (a red circle) appeared over 1 of the 12 search elements and disappeared within 100 ms in a shrinking fashion. The onset was presented randomly between one and three seconds from the start of the trial. This guaranteed that participants had commenced searching before the onset's appearance and allowed for sufficient time to react to the onset before the trial ended. Each trial lasted 4 s irrespective of the occurrence of a response. In between trials, a screen was presented for 1 s, showing only a fixation cross in the centre.

In different blocks of trials, four levels of congruency of the abrupt onset (0%, 10%, 50% and 100%) were tested. Congruency is defined as the spatial congruence of the target and onset stimuli. For example, during the 50% congruency block, the abrupt onset appeared on the target's location in half of the trials (congruent trails), whereas the abrupt onset appeared on a distracter's location on the remaining trials (incongruent trials). In addition to the four blocks with different onset congruencies, two control conditions were performed. In the first control condition, no onsets were presented. This condition was used to monitor gaze behaviour strictly related to the search task. In the second control condition (‘free’), participants were instructed to freely view the screen while an onset appeared. In this condition, all circles were closed and no target search had to be performed. This condition was administered to measure gaze capturing in the absence of a search task situation.

In total, each participant performed four sessions, with each session containing a total of six blocks (i.e. all four experimental conditions and the two control conditions). Each block contained 40 trials. Participants were offered to take a break in between sessions of up to 20 min. During the first and third session, the order of presentation was: no onset, 0%, 10%, 50%, 100% congruency, and free viewing. This order was reversed during the second and fourth session, to prevent performance bias due to learning or fatigue over conditions. Before the start of each block, a message on the screen informed participants about the upcoming condition (i.e. the congruency value, or an indication of the free viewing or the no-onset condition).

#### Instructions

Prior to the experiment, participants were instructed to search for the circle with just one opening during the search task and, when found, to report on which side it was open. They were required to do this as fast and as accurately as possible. Furthermore, participants were instructed to gaze freely over the screen in the free viewing condition and to fixate on the fixation cross in between trials. During instruction, stimulus screens were shown and participants completed a few practice trials so that they understood the task. Finally, participants were instructed to try to search for the target immediately after the start of the trial and not wait for the onset to appear. Participants were instructed that in some trials attending to the onsets could help finding the target, while in other trials attending to the onset would hinder finding the target.

### Analysis

#### Capture criteria

To measure whether participants' attention was captured, we analysed eye movements (saccades) and used oculomotor capture as a marker for attentional capture. A saccade was defined by a change in eye position of at least 1.0 deg, at a velocity of at least 30 degrees/s, and an acceleration of 8000 degrees/s/s. We applied the following criteria to classify a saccade as an oculomotor capture: (a) a saccade was made that landed on the location of onset appearance, and (b) it was the first or second saccade after the onset's appearance (we also analysed the second saccade because the onset could have been presented during the saccadic dead time of the first saccade made after the onset). A saccade was classified as having been made towards the onset, and thus as a capture, when its end position was closer to the location of the object where the onset appeared than to the location of any other object (i.e. the saccade had to land within a radius of 2.5–2.9 deg of an onset object). We disregarded saccades made earlier than 100 ms after onset appearance (to exclude saccades that were already programmed before the onset's appearance), as well as saccades with latencies larger than 1000 ms. In 24% of the trials the first saccade following onset appearance was within 100 ms. In these cases the next saccade for this trial was used, (i.e. the trial was not removed from analysis) In 5% of the trials, the first saccade following the onset appearance was later than 1000 ms; these trials were removed from further analysis.

#### Logistic regression to determine attentional window size

The oculomotor capture data can be used to determine the capture efficiency. In combination with logistic regression, the capture data was used to determine the attentional window size. An oculomotor capture was assigned a value of 1; an onset that did not capture gaze (a ‘miss’) was assigned a value of 0. We fitted a standard psychometric function (i.e., a sigmoid or S-curve) to these outcomes as a function of retinal eccentricity (i.e. the visual angle at which the onset appeared relative to the gaze position at that moment) [20]. We chose the eccentricity corresponding to 50% capture effectiveness (which is identical to capture frequency or capture likelihood) as a measure for the attentional window size. Three constraints were imposed while fitting the logistic function to obtain stable and realistic fits/outcomes. First, the logistic curve had to be descending. Second, the 50% capture point could not be negative (i.e. should be higher or equal to 0). Third, the lower and upper limits of the logistic curve (base and miss rates respectively) were allowed to vary between 0% and 5% however the variation must be equal in size (although in principle these can be independent, simultaneous adaptation assured that the curve's center remained at 50% capture effectiveness).

The psychometric functions were fitted in MATLAB. Fits were performed for each participant and for each condition. To evaluate the influence of chance in determining the size of the attentional window, data in the condition without an onset was analysed based on “virtual” onsets. For this, for each trial a random timing and onset location was chosen, in a way identical to that in actual onset conditions during the experiment. In all other ways, analysis was identical to the analysis of trials with actual onsets present.

Repeated measures ANOVA was used to determine an effect of condition on performance (target identification), reaction time, and saccadic latency (in case of captures in conditions with onsets). The relationship between capture effectiveness and attentional window size was investigated using a t-test for paired samples.

## Results

Congruency significantly affected search performance, i.e. the relative number of trials in which the side of the target containing the gap was correctly identified (*F* (4,52)  = 49.5, p<0.001) with higher performance for higher congruencies (data in table 1). In the conditions with higher congruencies, response times (*F* (4,52)  = 3.5, p = 0.014) were shorter (determined for correct responses). Congruency affected average saccadic latency (*F* (4,52)  = 17.9, p<0.001), with shorter latencies for higher congruency conditions. Latency in the “free” condition was somewhat comparable to that in the 50% congruency condition.

**Table 1:** Summary of results.

Condition	Performance	Response time (ms)	Saccadic latency (ms)
No onset	76.0 (3.3)	2278 (37)	n.a.
0% congruency	74.0 (3.2)	2304 (34)	414 (8)
10% congruency	75.4 (2.7)	2293 (35)	407 (7)
50% congruency	81.9 (2.4)	2290 (47)	377 (9)
100% congruency	98.4 (0.6)	2186 (46)	333 (10)
No search (‘free’)	n.a.	n.a.	376 (21)

Table lists mean search performance (correct identification of the direction of the target's gap), average response time for correct trials, and saccadic latencies for captures (standard errors over participants in brackets).

**Figure 2:**
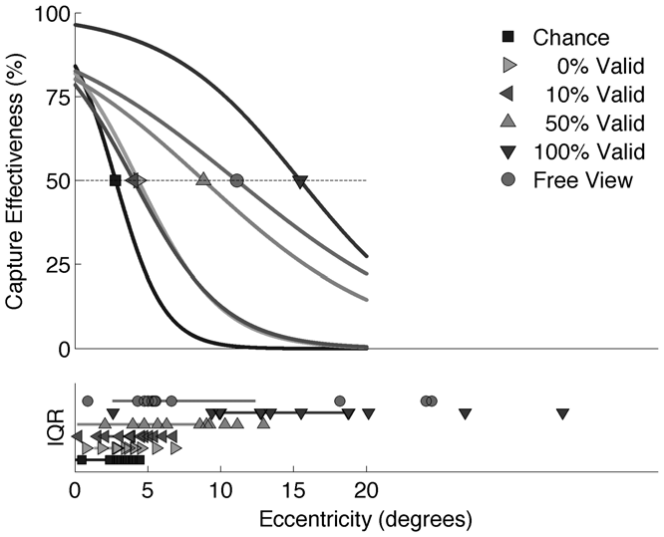
Upper graph: oculomotor capture effectiveness versus eccentricity of onset appearance. Capture effectiveness in the condition without an onset (‘Chance’) indicates the chance of visiting the onset's location regardless of the abrupt onset's influence. In this condition, no actual onset was presented (see method for details). The dotted line indicates the level of 50% capture effectiveness. The eccentricity at which each curve crosses this line represents the attentional window size. Lower graph: interquartile ranges of attentional window sizes over participants (lines), together with individual participants' attentional window sizes (symbols). Bootstrapped 90% confidence intervals yielded a similar spread. Goodness of fit information can be inferred from the interquartile ranges reported for each subject in each condition. They provide a confidence interval for the fitted attentional window width (at 50%). In addition, the interquartile ranges allow for assymmetry in the upper and lower bounds. (Free View: 2 subjects did not provide a good fit, 100%: 5 subjects did not provide a good fit because they did not show a decrease in capture efficiency with higher eccentricity.)

The main question of this paper concerns how congruency affects attentional window size. Figure 2 shows the sigmoid functions fitted to the capture data in order to quantify attentional window size. The data indicates that attentional window size increases with higher onset congruency. Attentional window size in the “free” condition was comparable to that in the 50% congruency condition. Paired t-tests comparing the eccentricity corresponding to 50% capture effectiveness (our measure of attentional window size) confirmed this impression. Attentional window size was larger in the 50% than in the 10% congruency condition (t = −4.2; p = 0.001), and also larger in the 100% than in the 50% congruency condition (t = −2.3; p = 0.05). The latter test presumably somewhat underestimates the magnitude and significance of the effect. In the 100% congruency condition, a sigmoid function could be reliably fitted to the data in only 10 of the 14 participants. Inspection of the data of the four participants to which no individual function could be fitted indicated that the reason for the ill-fitting was a very high capture effectiveness throughout the visual field. For these participants, the estimate of the attentional window size in the 100% congruency condition would therefore exceed the bounds of the current plot. (Note that this is not an issue in our estimate of the attentional window size based on the pooled data.)

To verify whether inhibition of return [21] had any significant influence on the results, we reanalysed the data excluding trials in which the onset location had already been visited prior to the capture taking place. Although this had a small influence on the positions of the curves, it did not affect the main effect, namely that attentional window size increased with congruency.

## Discussion

We found that in a search task, attentional capture, evident from explicit eye-movements towards an abrupt onset, occurred much more frequently during high-congruency onsets than during low-congruency onsets. This indicates that observers voluntarily modulate the size of their attentional window in accordance with the expected relevance of the onset. In turn, this suggests that voluntary modulation of attentional window size is a mechanism by which observers regulate whether or not environmental signals are allowed to capture their attention.

We used overt, oculomotor capture (i.e. capture of gaze) as our primary measure of attentional capture. Although conditions have been reported in which attentional and oculomotor capture are decoupled, during search for a unique shape (gap) as in our experiment, color singletons elicit both attentional capture and oculomotor capture [22]. Therefore, it seems justified to use oculomotor capture as an indicator of attentional capture and thus as a measure of attentional window size (see also [23] for a recent paper that also uses an oculomotor approach).

### Type of task and strategic search modes

That a adjustable spatial window of attention regulates the occurrence of automatic capture [24] goes not unchallenged. Bacon and Egeth [25] proposed that whether attention will be captured or not depends on the strategic search mode adopted by participants, a position recently confirmed by Leber and Egeth [19]. When in singleton detection mode, participants use a saliency-based strategy for searching for targets (“find whatever object is unique with respect to its environment”). When in feature search mode (“search the environment for objects with a particular feature of interest”), participants are able to avoid attentional capture by non-matching singletons. Feature search mode constitutes a top-down strategy that can also be employed during parallel search. The very notion of two search modes already challenges the concept of completely automatic capture. However, Theeuwes [24] claimed that participants adopt a-variable-attentional window during parallel search, and that this explains the observed selectivity. According to Theeuwes, parallel search with automatic attentional capture is used within the attentional window. Outside this window, capture is not automatic and display items can be ignored.

### Do our results favour one or the other account?

Our results are consistent with the idea that participants voluntarily modulated the size of a spatial window in order to regulate which onsets did result in a capture, and which ones did not. The incentive given in the present experiment was the known relevance of the onset for indicating the target position. In conditions with identical stimulus displays and onsets, varying the relevance was sufficient to induce participants to change the spatial region within which onsets resulted in captures or not. In that sense, our results are entirely consistent whit the attentional window account and can be considered as a direct demonstration of its existence. Moreover, our approach provides a new method to directly measure the size of the attentional window.

Accounts in terms of strategic search modes, such as those proposed by Bacon and Egeth , that predict more or less uniform changes in attentional capture over the full extent of the display, by themselves are insufficient to explain our present pattern of results. However, our results cannot fully indicate which search mode was used within the attentional window. The search task required participants to search for gaps in the O's that made up the stimulus display. This would clearly have to be based on feature search mode. However, onset items were red imploding circles, detection of which in itself may have been based on either feature or singleton search mode. Deciding between the search modes used would require variation in the types of onset presented in our paradigm.

Our paradigm differs from that usually taken to study the attentional window. For starters, rather than showing that observers can ignore captures when in a parallel search mode, we made sure that participants were engaged in serial search by using a search task. While the default expectation is that this would result in a narrow window, we found otherwise. Participants could select which onsets captured attention, even to the extent that their spatial window covered the whole visual display-thus mimicking parallel search. Clearly, despite the task, participants still had voluntary control over their attentional deployment.

### Attentional window as a voluntary top-down strategy

The idea that automatic versus top-down control is guided by the type of search task appears to be an oversimplification [17], [25]. In fact, our results imply that top-down attentional employment may even more flexible than hitherto thought. Participants were able to voluntarily vary their window size and use large window sizes even during a serial task. So, potentially, it appears that observers may even employ different attentional windows (or search modes) for different task aspects. Moreover, our data indicates that they can set the size of their attentional window according to their *behavioural* goals. This conclusion does not exclude that the attentional window can be narrow during serial search tasks. In fact, we show that it is possible when little to no benefit for the search task is expected from examining the onset positions. Narrowing one's attentional window appears to be a deliberate choice, since a reaction to abrupt onsets may distract from systematic scanning of the environment [15]. For instance, when searching for the prototypical face in a crowd, it is advantageous to prevent ones attention from being captured by salient objects, like billboards or passing traffic. Similarly, when searching for information on a webpage, it is desirable to be able to avoid looking at obtrusive banners.

Our finding that participants used their prior knowledge – of the congruency rates – to voluntary modulate capture is in line with recent results by Sayim et al. [23]. They found a decrease in attentional capture by onsets –that participants were instructed to ignore– when the probability of the appearance of such an onset increased. Sayim et al. concluded that attentional capture could be modulated through statistical learning [23]. However, the extent to which this modulation was voluntary cannot be concluded from their experiment. Also, it cannot be concluded whether the learning resulted in a modulation of a spatial window from their results.

Overall, our current results favour the attentional window account. Moreover, this account may be a good candidate to reconcile the results of previous work (e.g.[7], [9], [12], [12], [14], [17], [24,25] , and explains how the observer's behavioural goals, expectations, and the resultant spatial window determines whether attention will be captured or not.

### Limitations

An alternative explanation for the large attentional window in high-congruency conditions, is that observers may “give up” on the search task and instead convert to a parallel search strategy based upon “pop-out” of the onset. Hence, changing congruency may have induced a change in the task strategy itself. However, we deem this explanation unlikely. First, we should note that the search task itself did not change, only the degree to which information provided by the onset might be helpful for the task. Second, we already find an increase of the attentional window for the lower congruency conditions (increasing congruency from 10% to 50%). In the latter condition, a complete reliance on onset pop-out would still have been very ineffective.

In addition, the proposed top-down regulated attentional window account does not provide a complete account of all attentional capture behaviour. Folk, Remington and Johnston [26] found that when a search screen was preceded by an (ir-) relevant cue, singletons at the search screen only captured attention when they shared some feature with the cue (i.e. were contingent, like having the same color, or having similar onset behaviour). A windowing mechanism alone would most likely not be able to explain this result and other factors such as target characteristics may be important.

### Conclusion

Through measuring attentional capture using gaze tracking, we showed that the attentional window is modulated in accordance with the expected relevance of environmental signals. For this, we used an (ongoing) search task that enabled daily life visual behavior. The more relevant the signals were expected to be, the larger the observers set their attentional window. Thus, attentional window size can be voluntarily modulated in accordance with individual behavioural goals. This account of attentional capture may be applied to improve our ability to guide an observer's attention and gaze to regions of interest, with the ultimate goal of improving information communication.
